# Dimensions of the Complexity of Health Interventions: What Are We Talking about? A Review

**DOI:** 10.3390/ijerph17093069

**Published:** 2020-04-28

**Authors:** Justine Trompette, Joëlle Kivits, Laetitia Minary, François Alla

**Affiliations:** 1 EA4360, Université de Lorraine, 54000 Nancy, France; justine.trompette@univ-lorraine.fr (J.T.); joelle.kivits@univ-lorraine.fr (J.K.); laetitia.minary@univ-lorraine.fr (L.M.); 2 Bordeaux Population Health Research Center, UMR 1219, Inserm, Université de Bordeaux, 33000 Bordeaux, France

**Keywords:** complex intervention, health promotion, complexity, intervention, evaluation

## Abstract

Many recommendations and innovative approaches are available for the development and evaluation of complex health interventions. We investigated the dimensions of complexity described in health research and how these descriptions may affect the adopted research methodology (e.g., the choice of designs and methods). We used a mixed method approach to review the scientific literature evaluating complex interventions in the health field. Of 438 articles identified, 179 were subjected to descriptive analysis and 48 to content analysis. The three principal dimensions of complexity were: stakeholder characteristics, intervention multimodality and context. Recognition of such dimensions influenced the methodological choices made during evaluation of the interventions with their use of designs and methods, which aimed to address the complexity. We analysed not only how researchers view complexity but also the effects of such views on researcher practices. Our results highlight the need for clarification of what complexity means and to consider complexity when deciding how to evaluate research interventions.

## 1. Background

Complex interventions [1,2] challenge both researchers and stakeholders in terms of development and evaluation [3]. These interventions also pose challenges when transferring them to different contexts and scaling them up or out [4,5,6].

Given the substantial influences of intervention characteristics and context on the results [7,8], evaluating complex interventions involves more than assessing their effectiveness. The mechanisms, processes, conditions and modality of implementation must also be explored as to their impacts on context [9]. Researchers are effectively invited to open a “black box” that allows for the assessment of functionality, reliability, quality and causal mechanisms and contextual factors associated with variations in outcomes [10,11].

Several approaches to complexity have been put forward. In 2000, the Medical Research Council (MRC) proposed a definition (revised in 2008) of a complex health intervention [1,10]. Complexity involves the interaction of multiple components, the behaviour of those providing and receiving the intervention, the organisational level targeted, the variability in outcomes and the flexibility of the intervention. Another approach to analysing complexity uses complex-systems thinking [9,12]. Intervention/system complexity is defined in terms of its components and its associations with various systems. Many factors must be considered, including internal (e.g., activities, human, material and financial resources) and external factors (e.g., the social environment and political context). The intervention context embraces the spatial and temporal conjunction of social events, individuals and social interactions; these generate causal mechanisms that interact with the intervention and modify the intervention’s effects [13].

Recognising the system characteristics, Cambon et al. suggest, in their approach to complexity, replacing the term “intervention” with “intervention system” [14]. An intervention system was defined as a set of interrelated human and nonhuman contextual agents within certain spatial and temporal boundaries that generate mechanistic configurations (mechanisms) that are prerequisites for changes in health.

A previous review highlighted challenges imposed by the design, implementation and evaluation of complex interventions [3]. They have to do with the content and standardisation of interventions, the impact on the people involved, the organisational context of implementation, the development of outcome measures and evaluation. Here we focus on evaluation. We recently published a review of the principal methods used to evaluate complex interventions [15]. We found that several methods are used successively or together at various stages of evaluation. Most designs feature process analyses. To complete this quantitative analysis, we used a mixed-methods approach to investigate the dimensions of complexity described in health research and how these descriptions may affect the adopted research methodology. More precisely, our specific objectives were to answer the following questions: 1. What are the key dimensions of complexity described by researchers? 2. How are such complexity dimensions evaluated? 3. How do they implement existing recommendations on complex interventions evaluation? and 4. How do they justify their choices at each stage of the research (from intervention development to its transferability)? Our previous article discussed the concepts and methods used by researchers as described in reviewed articles, but only an in-depth analysis of the entire contents (including the discussions) would clarify how choices were made.

An understanding of researcher attitudes and practices will inform work in progress, in particular, the development of tailored tools for the development, analysis, adaptation, transfer and reporting of interventions [16,17,18,19,20].

## 2. Methods

We performed a review [21] of peer-reviewed scientific articles evaluating complex interventions in the field of health. The quantitative part of this review has already been published [15]. This article presents the qualitative part of this review. The research strategy was designed to identify articles written by authors who evaluate complex interventions in the field of health (clinical care and health services research, health promotion and prevention) [15]. We used the PRISMA check-list [22].

### 2.1. Search Strategy

We searched the PubMed database on MEDLINE. The 11-year search period (from 1 January 2004 to 31 December 2014) contained studies that integrated the first definitions of complexity by the MRC [10,12]. We searched for the keywords “complex intervention[s]” AND “evaluation” in the title, abstract and/or body of the article.

### 2.2. Article Selection

Included articles met the following criteria: 1. They were written in English or French; 2. They were research/evaluation articles, protocols or feasibility/pilot studies (we excluded reviews/meta-analyses, methodological articles, abstracts, book chapters, commentaries/letters, conference papers and other oral presentations and articles reviewing conceptual frameworks); 3. They evaluated a complex intervention; 4. They include a presentation of the dimensions of complexity.

Two reviewers working independently performed the initial title and abstract screening (to exclude articles that were deemed ineligible) followed by a detailed full-text screening of the remaining articles (to exclude those that did not meet all the inclusion criteria). Any disagreements were resolved via discussion with a third reviewer.

### 2.3. Data Extraction

All articles were independently read by two researchers. Any disagreements were resolved via discussion. Data were extracted using a grid including the following items: 1. Description of articles (title, author(s), country, date, journal, publication type, key words, discipline, publication field and objective of the article); 2. Definition and description of complexity (reference(s) used, dimension(s) of complexity identified by authors) and methods used to address complexity.

### 2.4. Data Analysis

The extracted articles were subjected to a two-step analysis. The first step was a descriptive analysis that identified any included references to complexity. The second involved a content analysis exploring the dimensions of complexity, the concordance between the dimensions highlighted by the authors and those of relevant citations, the level of detail in the provided descriptions of complexity and the influence of the consideration of complexity on the researchers’ methods. Two authors (J.T., J.K.) performed these analyses and cross-checked their results.

## 3. Results

**Figure 1:**
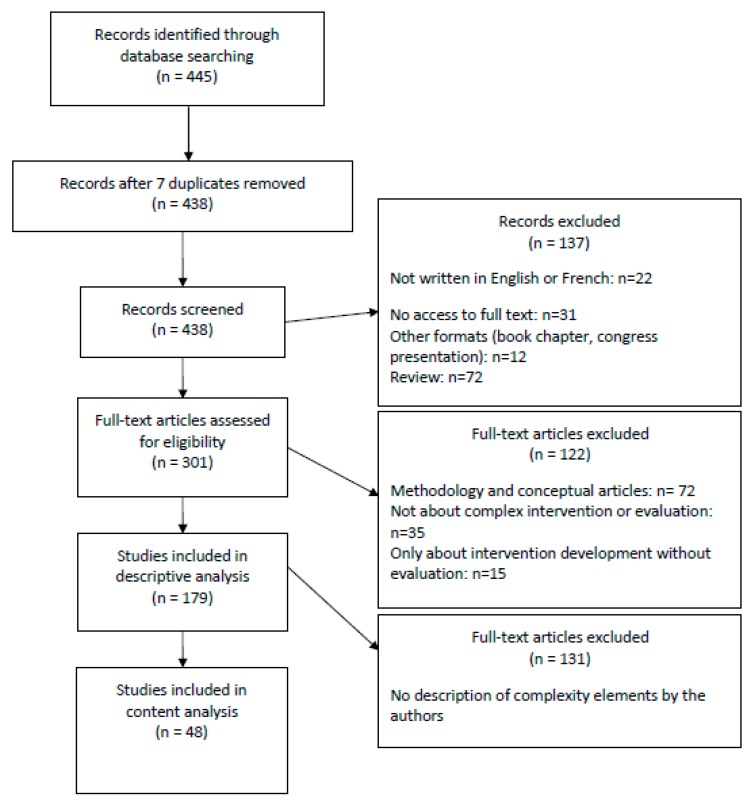
PRISMA flow chart.

A total of 438 articles were identified. Among them, 179 met the inclusion criteria (Figure 1).

### 3.1. General Description of the Published Articles

Of the 179 articles included in the study, 33.5% were protocols, 33.5% were pilot or feasibility studies and 33% were evaluation studies. Most articles originated from English-speaking countries. A tenfold increase in the number of articles on the broad theme of healthcare was observed over our screening period (from 4 in 2004 to 39 in 2014). Most articles involved researchers from several scientific disciplines (71.5%), representing collaboration between two (54.5%) to six (1.2%) disciplines. The most frequently represented disciplines were medicine, public health, social sciences, health sciences, nursing, psychology and health research. The articles dealt with the following fields: prevention, health promotion and education (46%), clinical care (36%) and health services research (18%). The main themes were healthcare (40%), pathology (33%) and behavioural change (27%).

Among the 179 articles, 52 did not use a reference involving complexity (the expression “complex intervention” appeared in the keywords, abstracts or body text but without any citation or description), 79 cited an article on complexity and 48 additionally defined, described or justified the intervention complexity. Among these 48 articles, the most frequently cited references (alone or combined) were the MRC frameworks of 2000 and 2008 and related articles [1,10] (86%), Campbell et al. [23] (15%), Oakley et al. [24] (12.5%), and Hawe et al. [12] (12.5%).

A content analysis of the 48 articles (Table 1 and Table 2) that went beyond a single citation allowed us to identify the dimensions of complexity in the interventions.

### 3.2. The Dimensions of Complexity

A thematic content analysis of the 48 articles revealed three major dimensions of complexity: 1. The characteristics of intervention stakeholders (27 articles); 2. The multimodality of an intervention (21 articles); and 3. The intervention context (14 articles).

#### 3.2.1. Characteristics of Intervention Stakeholders

A total of 27 articles cited the individual characteristics of the different stakeholders (beneficiaries, field workers and decision makers) as one of the dimensions of complexity. The individual characteristics reflected, on the one hand, both intervention stakeholders’ profiles and their possible effects in terms of intervention involvement and/or the relationships between the different stakeholders, and on the other hand, stakeholders’ knowledge and competencies (e.g., training). Although these combined characteristics were mentioned as elements of complexity, the individual characteristics were approached from different angles. Sometimes, they were treated as a “mechanism” or an “active ingredient” that was capable of positively or negatively influencing the outcome. For example, in article 45, authors explained that the complexity of their intervention, which was partly attributable to the professional competencies of the stakeholders, directly influenced the outcomes. At other times, the characteristics were treated as a research objective or as an outcome such as a behavioural change that was difficult to achieve:

*“Health care interventions […] are typically complex: they may […] seek to achieve outcomes that are difficult to influence, such as changes in individual patient or professional behaviour.”*

(article 8)

#### 3.2.2. The Multimodality of an Intervention

Multimodality was identified in 21 articles as a dimension of complexity. We found two types of multimodal interventions. Multimodality may be a combination of several very distinct actions (e.g., training professionals, environmental actions) performed simultaneously or consecutively at different levels. Alternatively, multimodality may be a chain of stages in a single action (e.g., a medical treatment protocol). In article 28, authors compared the results of the evaluation of a complex rehabilitation protocol to the usual treatments for patients discharged from intensive care units. The complexity here lay in a proposal to redesign care to feature several consecutive steps.

#### 3.2.3. The Intervention Context

The context in which the intervention was described was another dimension of complexity in 14 articles. Irrespective of whether the context was organisational, institutional, environmental, or social, it was cited because of its strong influence on both the implementation of the intervention (generally involving some flexibility) and its actual effectiveness.

In article 41, authors explained the importance of defining elements of the context and identifying how these might (positively or negatively) influence the outcomes.

*“To describe the contexts in which the intervention was delivered and explore contextual factors that may influence the delivery or impact of the intervention, and the outcomes. […] It may also explain intentional and unintentional differences in delivery. Reflecting on the relationship between organisational context and how each agency used the program to address local needs may have implications for future program design and delivery. […] We conceptualised context as incorporating the social, structural and political environment of each participating organisation.”*

(article 41)

Unlike the two previous dimensions, which, from the authors’ viewpoints, were sufficient to define interventions as complex, context was always combined with one or several other dimensions of complexity. In other words, context did not appear as an independent component, and, when it did appear, the complexity lay principally in the interaction between the context and stakeholders’ characteristics and/or the multimodality of the intervention.

### 3.3. The Level of Detail in Descriptions of Dimensions of Complexity

The level of detail varied greatly; we defined the approaches as minimalist, intermediate and in-depth descriptions. The minimalist approach corresponded to using only one citation for the source of complexity with little narrative description or details; the intermediate approach described a correspondence between the citation used and the elements of the intervention developed/evaluated; the in-depth approach included a structured and well-argued process of integrating dimensions of complexity into the evaluative approach.

#### 3.3.1. Minimalist Descriptions

Of the 48 articles analysed, 24 offered minimalist descriptions, mentioning one or more intervention components accompanied by a definition of complexity and a comment on whether this was relevant to intervention development or evaluation. At this level, the dimensions of complexity were identified in terms of the literature review only and not the precise intervention context. Intervention complexity was considered to justify an innovative evaluative approach, but the 24 articles each made only one “formal” link between the definition of complexity and their choice of methods for developing or evaluating their intervention.

#### 3.3.2. Intermediate Descriptions

Sixteen articles provided intermediate-level descriptions of complexity. Unlike in the Minimalist level discussed above, the authors did not simply identify the dimensions of the complexity of the intervention they were developing or evaluating based on the scientific literature, they also explained the dimensions by drawing on their own interventions.

Article 38 presented a pilot study of an e-intervention offering psycho-affective support from the siblings of people affected by their first psychotic episode. Authors introduced the notion of complexity via a citation and then emphasised the need to develop a more flexible, complex, multimodal intervention that could be adapted to the real lifestyles of their target population, whose dimensions of complexity they identified:

*“The intervention is “complex” according to the MRC definition because it comprises a number of, and interactions between, components within the intervention that subsequently impact on a number of variable outcomes. The components are: psychoeducation and peer support, each of which is a complex intervention in its own right, and each may work independently as well as inter-dependently in exerting their effects. […]. Another issue for consideration in complex interventions is the number and difficulty of performing behaviours required by those delivering and/or receiving the intervention, such as those involved in this intervention by the sibling-participants.”*

(article 38)

At this level, although a link was established between the reference(s)/concept(s) used and the elements present in the interventions, reflection on complexity and its elements remained rather limited.

#### 3.3.3. In-Depth Descriptions

The other eight articles provided detailed descriptions of complexity. These in-depth works deconstructed interventions into their various components to identify the dimensions of complexity and thus determine a corresponding methodology for intervention development and evaluation. The authors applied the concepts as closely as possible to their intervention practice. For example, a study on the dental health of patients who had suffered strokes structured complexity differently from that described above. Authors (article 16) interpreted their intervention theoretically by referencing the literature and deconstructing it into components that differed in terms of complexity. In their opinion, the extent of complexity depended on the number of interactions between levels, components and actions targeted by the intervention, the number and variability of participating groups and the contexts of the interventions.

The authors showed, on the one hand, a link between each element and the systems engaged and, on the other hand, the influence of such interdependence on the outcome. In other words, they formulated hypotheses of intervention mechanisms. They considered that an understanding of the interactions between the different elements was essential if a real world intervention was to be successful. This implied that these elements had been considered to matter when the research questions had been formulated and ceased only when the evaluative process was performed. Their analysis of complexity was integral to the development of the intervention, guiding first the theorisation and then the evaluative framework, allowing them to measure the impact of intervention components by the different contexts targeted.

Another article presented an exercise programme for community-dwelling, mobility-restricted, chronically ill older adults featuring structured support by general practitioners (article 34). The authors deconstructed the complexity of their intervention and developed a methodological strategy guided by the MRC framework. They combined all five of the MRC guidance dimensions with specific intervention elements. For example, for the item “number and difficulty of behaviours required by those delivering or receiving the intervention”, they identified “GPs’ counselling behaviour, exercise therapist’s counselling behaviour and patient’s behaviour including motivation and ability (information, instruction, equipment) to perform the exercise programme regularly”. They built their study via detailed reference to the first three phases of the MRC framework.

### 3.4. Recognising Complexity When Considering Interventions and Evaluations

In 30 of the articles (60.7%), intervention development and/or evaluation followed only the MRC recommendations. Thirteen (27.1%) cited Hawe et al. [12] and/or another reference specific to the theme of the intervention and five (10.4%) cited a complex intervention similar to that contemplated from within the authors’ own discipline.

Although all articles reported the use of methodological approaches that had been adapted to consider complexity, their application varied among studies, as the approaches were introduced at different points during the intervention. Of the 48 studies, 13 (27%) considered complexity only during the development of the intervention, 17 (35%) only during evaluation and 18 (37.5%) throughout the entire process.

Regardless of the point at which complexity was considered, such consideration caused 47 authors (98%) to use a methodological adaptation (e.g., a cluster randomized controlled trial (RCT)) or an alternative (e.g., realistic evaluation) to the individual RCT, the gold standard in clinical research. The principal adaptation was the creation of some form of process evaluation (46%), usually (but not always) combined with a trial. However, the descriptions of the methodological adaptations in relation to the dimensions of complexity varied in their level of detail, as did the reasons for the choices. Some authors cited only a single reference to the development and evaluation of complex interventions to justify the use of a particular methodology and/or evaluative model, specifically when that model diverged from the MRC recommendations. Others strictly applied the recommendations to which they were referring, or engaged in detailed reflection on their methodological choices in terms of the complexity of the intervention, also describing the adaptations in great detail. They also often advocated a wider use of the framework they had followed. In article 10, authors clearly justified their evaluative choices and sought to consider, optimally, the various dimensions of the complexity of their intervention.

*“This complexity makes a classical randomized controlled trial (RCT) design […] inappropriate for evaluating the effectiveness of public health interventions in a real-life setting [15,16]. […] Therefore, an evaluation approach is proposed that includes a combination of quantitative and qualitative evaluation methods to answer the three research questions of this study. To answer the first and second question, a quasi-experimental pre-test post-test study design […] is used. […] intervention inputs, activities, and outputs are recorded to assess the implementation process.“*

(article 10)

They returned to a discussion of the contributions made by their evaluative choice and explained that, in their opinion, a mixed-methods approach yielded detailed information on both the methodological feasibility of the intervention and its exact specification.

Finally, fewer than half of the articles (43%) used a theory-based approach to intervention development. The most frequently used theories were psychosocial constructs of behavioural change (21%).

## 4. Discussion

We analysed how health researchers defined and described complexity in their evaluations and how these descriptions influenced their methodological choices. This qualitative analysis complements our previous article on methodologies used [15]; here we seek to explain why they were used.

We found three principal dimensions of complexity:

1. Stakeholder characteristics were viewed as either a mechanism/active ingredient or an outcome to be achieved in terms of behavioural change. 2. The next dimension, multimodality, is key. However, multimodality per se does not adequately define complexity. In fact, a “complicated” intervention is distinguished from a “complex” one not only by the multimodality but also by its dynamic character and interactions with the context [25]. 3. The interaction of the intervention with the organisational, social, environmental, or institutional context was clearly recognised as constitutive of complexity. Our review of experiences and practices, as reported in the articles, emphasises the perceived importance of such interactions in terms of intervention effects. This is consistent with the view that a complex intervention is an “event in a system” [2,9,14]. Pragmatic operational approaches are being developed to consider context in developing, evaluating and adapting interventions [17,26,27].

Identifying dimensions of complexity contributes to a better understanding of how and under what conditions an outcome can be achieved. This approach is essential also for transferability as it helps to highlight the “key functions” that need to be maintained to obtain similar outcomes in a new setting [4].

Most identifications and explanations of complexity were pragmatic in nature, countering the ambiguity generated by semantic variations when describing the dimensions of complexity [28]. The authors rarely used recognised terms such as “active ingredients” [29], “key functions” [12], “core elements” [30] and even “components” [10,31]. Reporting of interventions requires improvement [20,32]. In fact, the descriptions of interventions, processes, mechanisms and theoretical models were often poor [32,33]. A detailed description may not be feasible in a scientific article, but articles that included either intermediate or in-depth descriptions tended to highlight just one component of intervention and used methodological adaptations for evaluation. Half of the articles engaged in process evaluation, usually coupled with a trial, to evaluate implementation quality and clarify the mechanisms that did or did not impact the expected intervention outcomes, thus conforming to the MRC recommendations [1,11]. However, any connection between the recognition of intervention complexity and the decision to adapt a certain evaluative method received minimal attention; complexity per se was generally viewed as adequate justification for the use of an evaluative model other than the classic individual RCT, which remained the gold standard, even for those authors who used methodological adaptations [15].

The descriptions of dimensions of complexity varied in terms of precision and depth, the methodologies used and the justifications for adaptations introduced because of complexity. The extent to which researchers and stakeholders deal with complexity differ, as does the correspondence between theory and practice. Indeed, not all intervention complexity dimensions defined in the literature were used, or even mentioned, by a number of authors who claimed that their interventions were complex. However, not all dimensions are always relevant for a given intervention/evaluation. Furthermore, the number of dimensions and the extent to which complexity is considered in a research project depends on many factors, such as the questions asked [15]. Although some new dimensions of complexity were identified, they were generally only briefly discussed. We cannot tell whether this was because the concepts had only been partially adopted by the researchers and field workers or because “theoreticians” and “practitioners” viewed complexity differently.

### Strengths and Limitations

The dimensions of complexity can be classified in various ways [11,34]. We have chosen a stakeholders/multimodality/context classification. This was appropriate because it reflected the concerns of the authors. This classification was pragmatic, not mechanistic. Other choices could have been made, e.g., the characteristics of those delivering interventions could have been viewed as either implementation elements and/or key functions (i.e., producers of the result).

We may not have retrieved every useful work on complex interventions. The keyword “complex intervention” retrieved only interventions described as complex by their authors. A previous review on the same subject used a similar method (a search for the term “complex intervention” in the title and/or abstract) [3]. Moreover, the PubMed database references articles in the field of health but lacks some in the fields of educational and social sciences. We did not seek to be exhaustive; rather, we sought to provide an overview of the links between definitions of complexity and choices of evaluation methodologies made by researchers who identify themselves in this way.

Finally, a review of the scientific literature cannot fully achieve the objectives that we set. A review can deal with the practices of only a small proportion of researchers and stakeholders.

## 5. Conclusions

Here we clarified not only how researchers view complexity but also the effects of such views on researcher practices. Our results highlight the need for clarifications of what complexity means. We would encourage researchers to pay careful attention to their descriptions of the dimensions of the complexity of the interventions they evaluate.

## Figures and Tables

**Table 1:** List of included articles.

N	Articles Included
1	Power R, Langhaug LF, Nyamurera T, Wilson D, Bassett MT, Cowan FM. Developing complex interventions for rigorous evaluation—a case study from rural Zimbabwe. Health Educ Res. 2004 Oct;19(5):570–575.
2	Byrne M, Cupples ME, Smith SM, Leathem C, Corrigan M, Byrne MC, et al. Development of a complex intervention for secondary prevention of coronary heart disease in primary care using the UK Medical Research Council framework. Am J Manag Care. 2006 May;12(5):261–266.
3	Sturt J, Whitlock S, Hearnshaw H. Complex intervention development for diabetes self-management. J AdvNurs. 2006 May;54(3):293–303.
4	Sturt J, Hearnshaw H, Farmer A, Dale J, Eldridge S. The Diabetes Manual trial protocol—a cluster randomized controlled trial of a self-management intervention for type 2 diabetes [ISRCTN06315411]. BMC Fam Pract. 2006;7:45.
5	Panella M, Marchisio S, Gardini A, Di Stanislao F. A cluster randomized controlled trial of a clinical pathway for hospital treatment of heart failure: study design and population. BMC Health Serv Res. 2007;7:179.
6	Paul G, Smith SM, Whitford D, O’Kelly F, O’Dowd T. Development of a complex intervention to test the effectiveness of peer support in type 2 diabetes. BMC Health Serv Res. 2007;7:136.
7	Protheroe J, Bower P, Chew-Graham C. The use of mixed methodology in evaluating complex interventions: identifying patient factors that moderate the effects of a decision aid. Fam Pract. 2007 Dec;24(6):594–600.
8	Redfern J, Rudd AD, Wolfe CDA, McKevitt C. Stop Stroke: development of an innovative intervention to improve risk factor management after stroke. Patient EducCouns. 2008 Aug;72(2):201–209.
9	Lenz M, Kasper J, Mühlhauser I. Development of a patient decision aid for prevention of myocardial infarction in type 2 diabetes—rationale, design and pilot testing. Psycho-Soc Med. 2009;6:Doc05.
10	deVlaming R, Haveman-Nies A, Van’t Veer P, de Groot LC. Evaluation design for a complex intervention program targeting loneliness in non-institutionalized elderly Dutch people. BMC Public Health. 2010;10:552.
11	Freund T, Wensing M, Mahler C, Gensichen J, Erler A, Beyer M, et al. Development of a primary care-based complex care management intervention for chronically ill patients at high risk for hospitalization: a study protocol. Implement Sci IS. 2010;5:70.
12	Lemmens KMM, Nieboer AP, Rutten-Van Mölken MPMH, van Schayck CP, Asin JD, Dirven JAM, et al. Application of a theoretical model to evaluate COPD disease management. BMC Health Serv Res. 2010;10:81.
13	Protheroe J, Blakeman T, Bower P, Chew-Graham C, Kennedy A. An intervention to promote patient participation and self-management in long term conditions: development and feasibility testing. BMC Health Serv Res. 2010;10:206.
14	Siddiqi K, Khan A, Ahmad M, Shafiq-ur-Rehman. An intervention to stop smoking among patients suspected of TB—evaluation of an integrated approach. BMC Public Health. 2010;10:160.
15	Vanhaecht K, Sermeus W, Peers J, Lodewijckx C, Deneckere S, Leigheb F, et al. The impact of care pathways for exacerbation of Chronic Obstructive Pulmonary Disease: rationale and design of a cluster randomized controlled trial. Trials. 2010;11:111.
16	Brady MC, Stott DJ, Norrie J, Chalmers C, St George B, Sweeney PM, et al. Developing and evaluating the implementation of a complex intervention: using mixed methods to inform the design of a randomised controlled trial of an oral healthcare intervention after stroke. Trials. 2011;12:168.
17	Glaucoma screening Platform Study group, Burr JM, Campbell MK, Campbell SE, Francis JJ, Greene A, et al. Developing the clinical components of a complex intervention for a glaucoma screening trial: a mixed methods study. BMC Med Res Methodol. 2011;11:54.
18	Greenhalgh T, Campbell-Richards D, Vijayaraghavan S, Collard A, Malik F, Griffin M, et al. New models of self-management education for minority ethnic groups: pilot randomized trial of a story-sharing intervention. J Health Serv Res Policy. 2011 Jan;16(1):28–36.
19	Hartveit M, Biringer E, Vanhaeht K, Haug K, Aslaksen A. The Western Norway mental health interface study: a controlled intervention trial on referral letters between primary care and specialist mental health care. BMC Psychiatry. 2011;11:177.
20	Hirsch O, Keller H, Krones T, Donner-Banzhoff N. Acceptance of shared decision making with reference to an electronic library of decision aids (arriba-lib) and its association to decision making in patients: an evaluation study. Implement Sci IS. 2011;6:70.
21	Treweek S, Ricketts IW, Francis J, Eccles M, Bonetti D, Pitts NB, et al. Developing and evaluating interventions to reduce inappropriate prescribing by general practitioners of antibiotics for upper respiratory tract infections: a randomised controlled trial to compare paper-based and web-based modelling experiments. Implement Sci IS. 2011;6:16.
22	Bath B, Pahwa P. A physiotherapy triage assessment service for people with low back disorders: evaluation of short-term outcomes. Patient Relat Outcome Meas. 2012 Jul;3:9–19.
23	Bench SD, Day TL, Griffiths P. Developing user centred critical care discharge information to support early critical illness rehabilitation using the Medical Research Council’s complex interventions framework. Intensive Crit Care Nurs Off J Br AssocCrit Care Nurses. 2012 Apr;28(2):123–131.
24	Chan CWH, Richardson A, Richardson J. Evaluating a complex intervention: a process evaluation of a psycho-education program for lung cancer patients receiving palliative radiotherapy. Contemp Nurse. 2012 Feb;40(2):234–244.
25	Cresswell KM, Sadler S, Rodgers S, Avery A, Cantrill J, Murray SA, et al. An embedded longitudinal multi-faceted qualitative evaluation of a complex cluster randomized controlled trial aiming to reduce clinically important errors in medicines management in general practice. Trials. 2012;13:78.
26	Irvine L, Falconer DW, Jones C, Ricketts IW, Williams B, Crombie IK. Can text messages reach the parts other process measures cannot reach: an evaluation of a behavior change intervention delivered by mobile phone? PloS One. 2012;7(12):e52621.
27	Jack SM, Ford-Gilboe M, Wathen CN, Davidov DM, McNaughton DB, Coben JH, et al. Development of a nurse home visitation intervention for intimate partner violence. BMC Health Serv Res. 2012;12:50.
28	Walsh TS, Salisbury LG, Boyd J, Ramsay P, Merriweather J, Huby G, et al. A randomised controlled trial evaluating a rehabilitation complex intervention for patients following intensive care discharge: the RECOVER study. BMJ Open. 2012;2(4).
29	Yam PS, Morrison R, Penpraze V, Westgarth C, Ward DS, Mutrie N, et al. Children, parents, and pets exercising together (CPET) randomised controlled trial: study rationale, design, and methods. BMC Public Health. 2012;12:208.
30	Chandler CI, DiLiberto D, Nayiga S, Taaka L, Nabirye C, Kayendeke M, et al. The PROCESS study: a protocol to evaluate the implementation, mechanisms of effect and context of an intervention to enhance public health centres in Tororo, Uganda. Implement Sci. 2013;8:113.
31	Clyne B, Bradley MC, Hughes CM, Clear D, McDonnell R, Williams D, et al. Addressing potentially inappropriate prescribing in older patients: development and pilot study of an intervention in primary care (the OPTI-SCRIPT study). BMC Health Serv Res. 2013;13:307.
32	Geaney F, Scotto Di Marrazzo J, Kelly C, Fitzgerald AP, Harrington JM, Kirby A, et al. The food choice at work study: effectiveness of complex workplace dietary interventions on dietary behaviours and diet-related disease risk - study protocol for a clustered controlled trial. Trials. 2013;14:370.
33	Higginson IJ, Koffman J, Hopkins P, Prentice W, Burman R, Leonard S, et al. Development and evaluation of the feasibility and effects on staff, patients, and families of a new tool, the Psychosocial Assessment and Communication Evaluation (PACE), to improve communication and palliative care in intensive care and during clinical uncertainty. BMC Med. 2013;11:213.
34	Hinrichs T, Brach M, Bucchi C, Moschny A, Wilm S, Thiem U, et al. An exercise programme for community-dwelling, mobility-restricted and chronically ill older adults with structured support by the general practitioner’s practice (HOMEfit). From feasibility to evaluation. Z FürGerontolGeriatr. 2013 Jan;46(1):56, 58–63.
35	Mars T, Ellard D, Carnes D, Homer K, Underwood M, Taylor SJC. Fidelity in complex behaviour change interventions: a standardised approach to evaluate intervention integrity. BMJ Open. 2013;3(11):e003555.
36	Poston L, Briley AL, Barr S, Bell R, Croker H, Coxon K, et al. Developing a complex intervention for diet and activity behaviour change in obese pregnant women (the UPBEAT trial); assessment of behavioural change and process evaluation in a pilot randomised controlled trial. BMC Pregnancy Childbirth. 2013 Jul 15;13(1):148.
37	Round J, Drake R, Kendall E, Addicott R, Agelopoulos N, Jones L. Evaluating a complex system-wide intervention using the difference in differences method: the Delivering Choice Programme. BMJ Support Palliat Care. 2013 Aug 27;bmjspcare-2012-000285.
38	Sin J, Henderson C, Pinfold V, Norman I. The E Sibling Project—exploratory randomised controlled trial of an online multi-component psychoeducational intervention for siblings of individuals with first episode psychosis. BMC Psychiatry. 2013;13:123.
39	Yuen WW-Y, Wong WC-W, Tang CS-K, Holroyd E, Tiwari AF-Y, Fong DY-T, et al. Evaluating the effectiveness of personal resilience and enrichment programme (PREP) for HIV prevention among female sex workers: a randomised controlled trial. BMC Public Health. 2013 Jul 26;13:683.
40	Ettema R, Schuurmans MJ, Schutijser B, van Baar M, Kamphof N, Kalkman CJ. Feasibility of a nursing intervention to prepare frail older patients for cardiac surgery: a mixed-methods study. Eur J CardiovascNurs J Work Group CardiovascNursEurSocCardiol. 2015 Aug;14(4):342–351.
41	Haynes A, Brennan S, Carter S, O’Connor D, Schneider CH, Turner T, et al. Protocol for the process evaluation of a complex intervention designed to increase the use of research in health policy and program organisations (the SPIRIT study). Implement Sci IS. 2014;9:113.
42	Kwamie A, van Dijk H, Agyepong IA. Advancing the application of systems thinking in health: realist evaluation of the Leadership Development Programme for district manager decision-making in Ghana. Health Res Policy SystBioMed Cent. 2014;12:29.
43	Lawton R, Mceachan R, Jackson C, West R, Conner M. Intervention fidelity and effectiveness of a UK worksite physical activity intervention funded by the BUPA Foundation, UK. Health Promot Int. 2015 Mar;30(1):38–49.
44	Leamy M, Clarke E, Le Boutillier C, Bird V, Janosik M, Sabas K, et al. Implementing a complex intervention to support personal recovery: a qualitative study nested within a cluster randomised controlled trial. PloS One. 2014;9(5):e97091.
45	Leo SD, Bono L, Romoli V, West E, Ambrosio R, Gallucci M, et al. Implementation of the Liverpool Care Pathway (LCP) for the dying patient in the inpatient hospice setting: development and preliminary assessment of the Italian LCP program. Am J HospPalliat Care. 2014 Feb;31(1):61–68.
46	Nayiga S, DiLiberto D, Taaka L, Nabirye C, Haaland A, Staedke SG, et al. Strengthening patient-centred communication in rural Ugandan health centres: A theory-driven evaluation within a cluster randomized trial. EvalLondEngl 1995. 2014 Oct;20(4):471–491.
47	Rees K, Zweigenthal V, Joyner K. Implementing intimate partner violence care in a rural sub-district of South Africa: a qualitative evaluation. Glob Health Action. 2014;7:24588.
48	Watson DP, Young J, Ahonen E, Xu H, Henderson M, Shuman V, et al. Development and testing of an implementation strategy for a complex housing intervention: protocol for a mixed-methods study. Implement Sci IS. 2014;9:138.

**Table 2:** Description of included articles.

N	Reference	Description of Complexity	Refence(s) to Complexity	Part of Article Where Complexity Is Identified	Influence of Complexity Identification in Development/Evaluation of the Intervention	Type of Study	Evaluation Approach	Methodology Approach
1	Power R 2004	Minimalist	Campbell 2000	Background	In evaluation	Pilot study	- Quasi experimental trial- Process evaluation	Qualitative
2	Byrne M 2006	Minimalist	- Bradley 1999 - Medical research Council (MRC) 2000 - Campbell 2000 - Michie 2004 - Rowlands 2005 - Wong 2005	Background	In development	Pilot study	Formative/developmental evaluation	Mixed
3	Sturt J, J AdvNurs. 2006	Minimalist	MRC 2000	Background Methods	- In development - In evaluation	Pilot study	- Quasi experimental trial - Formative/developmental evaluation	Mixed
4	Sturt J, BMC Fam Pract. 2006	Minimalist	MRC 2000	Discussion	No	Evaluation	- RCT Cluster randomized controlled trial (RCT) - Process evaluation	Mixed
5	Panella M, 2007	Intermediate	- Campbell 2000 - Hawe 2004	Discussion	No	Evaluation	- Cluster RCT - Process evaluation	Quantitative
6	Paul G, S2007	Minimalist	- MRC 2000 - Campbell 2000 - Van-Meijel 2004	Background Discussion	In development	Pilot study	Formative evaluation/development	Mixed
7	Protheroe J, 2007	Intermediate	- MRC 2000 - Campbell 2000 - Oakley 2006 - Campbell 2007 - Vanhaecht 2007 - Panella 2009	Background	In evaluation	Evaluation	- Pragmatic trial - Process evaluation	Mixed
8	Redfern J, 2008	Intermediate	Campbell 2000	Background Methods Results Discussion	- In development - In evaluation	Pilot study	- Formative/developmental evaluation - Process evaluation	Mixed
9	Lenz M, 2009	Intermediate	- Campbell 2000 - Campbell 2007 - Lenz 2007 - Craig 2008	Methods Discussion	- In development - In evaluation	Pilot study	Formative /developmental evaluation	Qualitative
10	deVlaming R, 2010	Intermediate	- MRC 2000 - Craig 2008	Background Discussion	In evaluation	Evaluation	- Quasi experimental trial - Process evaluation	Mixed
11	Freund T, 2010	Minimalist	- Campbell 2000 - Campbell 2007 - Craig 2008	Background Methods	In development	Feasibility study	Formative/developmental evaluation	Mixed
12	Lemmens KMM, 2010	Intermediate	- Campbell 2000 - Campbell 2007 - Epping-Jordan 2005 - May 2006 - Lemmens 2008	Background Methods	In evaluation	Evaluation	- Quasi experimental trial - Process evaluation	Quantitative
13	Protheroe J, 2010	Minimalist	MRC 2000	Background Discussion	In development	Pilot study	Formative/developmental evaluation	Qualitative
14	Siddiqi K, 2010	Intermediate	- MRC 2000 - Campbell 2000	Background Methods	- In development - In evaluation	Pilot study Evaluation	- Cluster RCT - Process evaluation	Mixed
15	Vanhaecht K, 2010	Minimalist	- Campbell 2000 - Hawe 2004 - Oakley 2006 - Vanhaecht 2007 - Craig 2008 - Panella 2009 - Panella 2010	Background Methods Discussion	No	Evaluation	- Cluster RCT - Realistic evaluation - Process evaluation	Mixed
16	Brady MC, 2011	In-depth	- MRC 2008 - Shiell 2008	Background Methods Discussion	- In development - In evaluation	Pilot study	- Quasi experimental trial - Formative/developmental evaluation	Mixed
17	Burr JM, 2011	Minimalist	Craig 2008	Background Discussion	In development	Pilot study	Formative/developmental evaluation	Mixed
18	Greenhalgh T, 2011	Intermediate	- Hawe 2004 - Craig 2008	Background Discussion	- In development - In evaluation	Feasibility study	- RCT - Process evaluation	Mixed
19	Hartveit M, 2011	Intermediate	- MRC 2000 - Campbell 2000 - Craig 2008 - Vanhaecht 2011	- Background Methods Discussion	- In development - In evaluation	Pilot study	- Quasi experimental trial - Formative/developmental evaluation	Mixed
20	Hirsch O, 2011	Minimalist	- Craig 2008 - Protheroe 2007	Background Methods	In evaluation	Pilot study	Process evaluation	Mixed
21	Treweek S, 2011	Minimalist	MRC 2008	Background Discussion	In evaluation	Feasibility study	- RCT - process evaluation	Mixed
22	Bath B, 2012	Minimalist	Campbell 2000	Background Discussion	No	Pilot study	Quasi-experimental trial	Mixed
23	Bench SD, 2012	Minimalist	Campbell 2007	Background Methods Discussion	- In development - In evaluation	Pilot study	- Pragmatic and clustered trial - Formative/developmental evaluation	Mixed
24	Chan CWH, 2012	Intermediate	Campbell 2000	Background	In evaluation	Evaluation	- Pragmatic trial - Process evaluation	Mixed
25	Cresswell KM, 2012	Minimalist	- Bradley 1999 - MRC 2000 - Campbell 2000 - NIH 2004 - VanMeijel 2004 - Lindsay 2004 - Lewin 2009	Background Discussion	In evaluation	Evaluation	- Pragmatic and clustered trial - Process evaluation	Mixed
26	Irvine L, 2012	Minimalist	Oakley 2006	Background	- In development - In evaluation	Pilot study	- RCT - process evaluation	Mixed
27	Jack SM, 2012	Minimalist	- Campbell 2000 - Van-Meijel 2004 - Ford-Gilboe 2011	Background Results Discussion	In development	Feasibility study	Process evaluation	Qualitative
28	Walsh TS, 2012	In-depth	- Campbell 2000 - MRC 2000	Methods	In evaluation	Evaluation	- RCT - process evaluation	Mixed
29	Yam PS, 2012	Minimalist	Craig 2008	Background Methods	In development - In evaluation	Evaluation	- RCT - process evaluation	Mixed
30	Chandler CI, 2013	Minimalist	- Chen 1989 - Weiss 1995 - Power 2004 - Stame 2004 - MRC 2008 - Lewin 2009 - Bonell 2012 - Marchal 2012	Methods	In evaluation	Evaluation	- Cluster RCT - Process evaluation	Mixed
31	Clyne B, 2013	In-depth	- MRC 2000 - Grimshaw 2001 - Majumdar 2003 - Spinewine 2007 - Campbell 2007 - Craig 2008 - Kaur 2009 - Marcum 2010 - Loganathan 2011	Background Methods Results Discussion	In development	Pilot study	Formative/developmental evaluation	Qualitative
32	Geaney F, 2013	In-depth	Craig 2008	Background Methods	- In development - In evaluation	Evaluation	- Cluster RCT - Process evaluation	Mixed
33	Higginson IJ2013	Minimalist	Craig 2008	Methods	In development and evaluation	Pilot study	Formative/developmental evaluation	Mixed
34	Hinrichs T, 2013	In-depth	- Campbell 2000 - Chen 2005 - Campbell 2007 - Craig 2008 - Thabane 2010	Background Methods Results Discussion	- In development - In evaluation	Pilot study	Formative/developmental evaluation	Mixed
35	Mars T, 2013	In-depth	- MRC 2008 - Craig 2008	Background Discussion	- In development - In evaluation	Evaluation	- Pragmatic trial - Process evaluaiton	Quantitative
36	Poston L, 2013	Minimalist	Craig 2008	Background Discussion	In evaluation	Pilot study	-RCT - Evaluation process	Mixed
37	Round J, 2012	Minimalist	MRC 2008	Background Discussion	In development	Evaluation	- Quasi experimental trial - Process evaluation	Mixed
38	Sin J, 2013	Intermediate	- Lancaster 2004 - MRC 2008	Methods	- In development - In evaluation	Pilot study	RCT - Formative/developmental evaluation Process evaluation	Mixed
39	Yuen WW-Y, 2013	Minimalist	MRC 2008	Methods	In evaluation	Evaluation	RCT Evaluation process	Mixed
40	Ettema R, 2015	Minimalist	MRC 2008	Background Methods	- In development - In evaluation	Pilot study	Process evaluation	Mixed
41	Haynes A, 2014	Intermediate	- Harachi 1999 - Sanderson 2000 - Oakley 2006 - Grimshaw 2007 - MRC 2008 - Shiell 2008 - Hawe 2009 - Bradley 2011 - Ling 2012 - Moore 2013 - Brennan 2013	Background Methods Discussion	In evaluation	Evaluation	- Cluster RCT Process evaluation	Mixed
42	Kwamie A, 2014	In-depth	- Pawson 1997 - Begun 2003 - Holland 2006 - Sterman 2006 - Foster-Fishman 2007 - De Savigny 2009 - Hawe 2009 - Frenk 2010 - Prashanth 2012	Background Methods Discussion	In evaluation	Evaluation	Realistic evaluation -Process evaluation	Qualitative
43	Lawton R, 2015	Minimalist	- Dane 1998 - Hawe 2004 - Bellg 2004 - Oakley 2006 - Craig 2008	Background Discussion	In evaluation	Evaluation	- Cluster RCT Process evaluation	Mixed
44	Leamy M, 2014	Intermediate	- MRC 2000 - MRC 2008 - Thompson 2009 - Datta 2013	Background Methods Discussion	In evaluation	Evaluation	- Cluster RCT Process evaluation	Mixed
45	Leo SD, 2014	Intermediate	- Campbell 2000 - Campbell 2007	Methods Discussion	- In development - In evaluation	Pilot study	Formative/developmental evaluation	Mixed
46	Nayiga S, 2014	Intermediate	- MRC 2000 - Hawe 2004 - Oakley 2006 - Cartwright 2011 - English 2011	Methods	In evaluation	Evaluation	- Cluster RCT Process evaluation	Mixed
47	Rees K, 2014	Minimalist	Atun 2010	Methods Discussion	In evaluation	Feasibility study	Process evaluation	Qualitative
48	Watson DP, 2014	Minimalist	May 2006	Background Methods	No	Feasibility study	Process evaluation	Mixed

## References

[1] Craig P., Dieppe P., Macintyre S., Michie S., Nazareth I., Petticrew M. (2008). Medical Research Council Guidance. Developing and evaluating complex interventions: The new Medical Research Council guidance. BMJ.

[2] Hawe P., Shiell A., Riley T. (2009). Theorising interventions as events in systems. Am. J. Community Psychol..

[3] Datta J., Petticrew M. (2013). Challenges to Evaluating Complex Interventions: A Content Analysis of Published Papers. BMC Public Health.

[4] Cambon L., Minary L., Ridde V., Alla F. (2012). Transferability of interventions in health education: A review. BMC Public Health.

[5] Wang S., Moss J.R., Hiller J.E. (2006). Applicability and transferability of interventions in evidence-based public health. Health Promot. Int..

[6] Aarons G.A., Sklar M., Mustanski B., Benbow N., Brown C.H. (2017). “Scaling-out” evidence-based interventions to new populations or new health care delivery systems. Implement. Sci..

[7] McCabe S.E., West B.T., Veliz P., Frank K.A., Boyd C.J. (2014). Social contexts of substance use among U.S. high school seniors: A multicohort national study. J. Adolesc. Health.

[8] Shoveller J., Viehbeck S., Di Ruggiero E., Greyson D., Thomson K., Knight R. (2016). A critical examination of representations of context within research on population health interventions. Crit. Public Health.

[9] Shiell A., Hawe P., Gold L. (2008). Complex interventions or complex systems? Implications for health economic evaluation. BMJ.

[10] Campbell M., Fitzpatrick R., Haines A., Kinmonth A.L., Sandercock P., Spiegelhalter D., Tyrer P. (2000). Framework for design and evaluation of complex interventions to improve health. BMJ.

[11] Moore G.F., Audrey S., Barker M., Bond L., Bonell C., Hardeman W., Moore L., O’Cathain A., Tinati T., Wight D. (2015). Process evaluation of complex interventions: Medical Research Council guidance. BMJ.

[12] Hawe P., Shiell A., Riley T. (2004). Complex interventions: How “out of control” can a randomised controlled trial be?. BMJ.

[13] Poland B., Frohlich K.L., Cargo M., Potvin L., McQueen D.V., Hall M., Anderson L.M. (2008). Context as a Fundamental Dimension of Health Promotion Program Evaluation.

[14] Cambon L., Terral P., Alla F. (2019). From intervention to interventional system: Towards greater theorization in population health intervention research. BMC Public Health.

[15] Minary L., Trompette J., Kivits J., Cambon L., Tarquinio C., Alla F. (2019). Which design to evaluate complex interventions? Toward a methodological framework through a systematic review. BMC Med. Res. Methodol..

[16] Petticrew M., Knai C., Thomas J., Rehfuess E.A., Noyes J., Gerhardus A., Grimshaw J.M., Rutter H., McGill E. (2019). Implications of a complexity perspective for systematic reviews and guideline development in health decision making. BMJ Glob. Health.

[17] Pfadenhauer L.M., Gerhardus A., Mozygemba K., Lysdahl K.B., Booth A., Hofmann B., Wahlster P., Polus S., Burns J., Brereton L. (2017). Making sense of complexity in context and implementation: The Context and Implementation of Complex Interventions (CICI) framework. Implement. Sci..

[18] O’Cathain A., Croot L., Duncan E., Rousseau N., Sworn K., Turner K.M., Yardley L., Hoddinott P. (2019). Guidance on how to develop complex interventions to improve health and healthcare. BMJ Open.

[19] Flemming K., Booth A., Garside R., Tunçalp Ö., Noyes J. (2019). Qualitative evidence synthesis for complex interventions and guideline development: Clarification of the purpose, designs and relevant methods. BMJ Glob. Health.

[20] Candy B., Vickerstaff V., Jones L., King M. (2018). Description of complex interventions: Analysis of changes in reporting in randomised trials since 2002. Trials.

[21] Government of Canada, Canadian Institutes of Health Research (2010). (2010) A Guide to Knowledge Synthesis. CIHR. http://www.cihr-irsc.gc.ca/e/41382.html.

[22] Moher D., Liberati A., Tetzlaff J., Altman D.G. (2009). Preferred reporting items for systematic reviews and meta-analyses: The PRISMA statement. BMJ.

[23] Campbell N.C., Murray E., Darbyshire J., Emery J., Farmer A., Griffiths F., Guthrie B., Lester H., Wilson P., Kinmonth A.L. (2007). Designing and evaluating complex interventions to improve health care. BMJ.

[24] Oakley A., Strange V., Bonell C., Allen E., Stephenson J. (2006). RIPPLE Study Team. Process evaluation in randomised controlled trials of complex interventions. BMJ.

[25] Cohn S., Clinch M., Bunn C., Stronge P. (2013). Entangled complexity: Why complex interventions are just not complicated enough. J. Health Serv. Res. Policy.

[26] Craig P., Di Ruggiero E., Frohlich K.L., Mykhalovskiy E., White M. (2018). On behalf of the Canadian Institutes of Health Research (CIHR)–National Institute for Health Research (NIHR) Context Guidance Authors Group. Taking Account of Context in Population Health Intervention Research: Guidance for Producers, Users and Funders of Research.

[27] Movsisyan A., Arnold L., Evans R., Hallingberg B., Moore G., O’Cathain A., Pfadenhauer L.M., Segrott J., Rehfuess E. (2019). Adapting evidence-informed complex population health interventions for new contexts: A systematic review of guidance. Implement. Sci..

[28] Minary L., Alla F., Cambon L., Kivits J., Potvin L. (2018). Addressing complexity in population health intervention research: The context/intervention interface. J. Epidemiol. Community Health.

[29] Durlak J.A. (1998). Why Program Implementation Is Important. J. Prev. Interv. Community.

[30] Galbraith J.S., Herbst J.H., Whittier D.K., Jones P.L., Smith B.D., Uhl G., Fisher H.H. (2011). Taxonomy for strengthening the identification of core elements for evidence-based behavioral interventions for HIV/AIDS prevention. Health Educ. Res..

[31] Clark A.M. (2013). What Are the Components of Complex Interventions in Healthcare? Theorizing Approaches to Parts, Powers and the Whole Intervention. Soc. Sci. Med..

[32] Hoffmann T.C., Glasziou P.P., Boutron I., Milne R., Perera R., Moher D., Altman D.G., Barbour V., Macdonald H., Johnston M. (2014). Better reporting of interventions: Template for intervention description and replication (TIDieR) checklist and guide. BMJ.

[33] Michie S., Wood C.E., Johnston M., Abraham C., Francis J.J., Hardeman W. (2015). Behaviour change techniques: The development and evaluation of a taxonomic method for reporting and describing behaviour change interventions (a suite of five studies involving consensus methods, randomised controlled trials and analysis of qualitative data). Health Technol. Assess..

[34] Lewin S., Hendry M., Chandler J., Oxman A.D., Michie S., Shepperd S., Reeves B.C., Tugwell P., Hannes K., Rehfuess E.A. (2017). Assessing the complexity of interventions within systematic reviews: Development, content and use of a new tool (iCAT_SR). BMC Med. Res. Methodol..

